# Niche partitioning in a guild of invasive mammalian predators

**DOI:** 10.1002/eap.2566

**Published:** 2022-03-24

**Authors:** Patrick M. Garvey, Alistair S. Glen, Mick N. Clout, Margaret Nichols, Roger P. Pech

**Affiliations:** ^1^ Manaaki Whenua – Landcare Research Lincoln New Zealand; ^2^ Manaaki Whenua – Landcare Research Auckland New Zealand; ^3^ Centre for Biodiversity and Biosecurity, School of Biological Sciences University of Auckland Auckland New Zealand; ^4^ Centre for Wildlife Management and Conservation Lincoln University Canterbury New Zealand

**Keywords:** Carnivora, community ecology, food web, interference competition, invasive species, mustelid, niche differentiation, wildlife management

## Abstract

Predators compete aggressively for resources, establishing trophic hierarchies that influence ecosystem structure. Competitive interactions are particularly important in invaded ecosystems where introduced predators can suppress native prey species. We investigated whether niche partitioning exists within a guild of invasive mammalian predators and determined the consequences for native species. Over 4405 camera‐trap days, we assessed interactions among three invasive predators: two apex predators (feral cats *Felis catus* and ferrets *Mustela furo*) and a mesopredator (stoats *Mustela erminea*), in relation to their primary prey (lagomorphs, rodents and birds) and habitat use. Further, we tested for mesopredator release by selectively removing cats and ferrets in a pulse perturbation experiment. We found compelling evidence of niche partitioning; spatiotemporal activity of apex predators maximized access to abundant invasive prey, with ferrets targeting lagomorphs and cats targeting rodents. Mesopredators adjusted their behavior to reduce the risk of interference competition, thereby restricting access to abundant prey but increasing predation pressure on diurnal native birds. Stoats were only recorded at the treatment site after both larger predators were removed, becoming the most frequently detected predator at 6 months post‐perturbation. We suggest there is spatial and resource partitioning within the invasive predator guild, but that this is incomplete, and avoidance is achieved by temporal partitioning within overlapping areas. Niche partitioning among invasive predators facilitates coexistence, but simultaneously intensifies predation pressure on vulnerable native species.

## INTRODUCTION

Predators are an important driving force in ecosystems, regulating food webs and the distribution of resources (Terborgh & Estes, 2010). Apex predators suppress subordinate mesopredators directly through agonistic encounters or indirectly by reducing resource availability (Ritchie & Johnson, 2009). Mesopredators, in turn, reduce the risk of agonistic encounters by deviating from an optimum resource gathering strategy (Pianka, 1974; Ritchie & Johnson, 2009). This process of partitioning resources, known as niche differentiation, prevents the extirpation of the subordinate predator, as would otherwise be predicted by the competitive exclusion principle (Schoener, 1974).

Niche partitioning by coexisting predators can occur in time, space, and resources. Temporal partitioning can reduce both resource and interference competition, regardless of which form of competition was the catalyst for these changes (Kronfeld‐Schor & Dayan, 1999). Dominant predators preferentially occupy the most productive areas, leaving less productive areas for subordinate species, leading to spatial partitioning (Durant, 2000). For example, least weasels (*Mustela nivalis*) exhibit a reciprocal distribution with stoats (*M. erminea*) in parts of their native range, being rare or absent in optimal prey habitats, but occurring more frequently in less productive areas (Erlinge & Sandell, 1988). Coexistence may also be facilitated by the partitioning of prey species (McDonald, 2002), prey size (Sinclair et al., 2003) or by morphological differences adapted for prey selection (Krebs, 1978). Where considerable overlap occurs in one niche dimension, differences should occur in other dimensions (Pianka, 1974).

Mammalian predators are among the most damaging invasive species in the world, responsible for 58% of all known bird, mammal and reptile extinctions (Doherty et al., 2016; Terborgh & Estes, 2010; Veale et al., 2015). Negative impacts can be exacerbated when invasive predators interact with other threats (Doherty et al., 2015). The remote islands of New Zealand, where native species have evolved in isolation for more than 70 million years, provide invasive mammalian predators with ideal ‘niche opportunities’: plentiful prey, no native competitors, few mammalian diseases and parasites, and a heterogeneous environment likely to promote coexistence with other invasive predators. Feral cats (*Felis catus*), ferrets (*M. furo*), and stoats are the largest introduced predators and are responsible for declines and extinctions of many native species (Parkes & Murphy, 2003). The competitive hierarchy among New Zealand's largest introduced predators remains unclear; cats are predicted to dominate ferrets in competitive interactions, given the propensity of felids to engage in interspecific killing and by virtue of their larger size (cat = ~3 × ferret) (Donadio & Buskirk, 2006; Palomares & Caro, 1999). However, the literature supplies scant evidence to support this; for example, ferrets maintained possession or displaced cats during seven out of eight interactions recorded at animal carcasses (Ragg et al., 2000). Stoats are the subordinate member of this guild; cats and ferrets dominate through interference competition (Garvey et al., 2015) and intraguild predation (Wodzicki, 1950). Fear of dominant carnivores also imposes fitness constraints, influencing stoats’ foraging and vigilance behaviors (Garvey et al., 2015).

Our study objective was to evaluate the roles of top‐down (interference competition) and bottom‐up (prey availability, habitat structure) processes in determining niche partitioning and intraguild interactions. In New Zealand, invasive predators depend on other introduced pests as their primary prey, a resource that fluctuates due to natural or management‐driven perturbations (Norbury, 2017). In turn, interspecific competition among invasive predators will affect their distribution and demography, leading to niche divergence or competitive exclusion. Understanding the relative importance of top‐down versus bottom‐up forces will allow wildlife managers to predict the consequences of pest removal and avoid perverse outcomes, such as mesopredator release. Even when an adaptive management strategy is implemented, differences in individual traits, trapability, succession, and recolonization rates among invasive predators imply that understanding the drivers of niche partitioning warrants consideration (Garvey et al., 2015; Garvey et al., 2020).

There is a lack of experimental studies on niche partitioning within carnivore guilds, due to the ethical and logistical constraints of manipulative experiments (Sévêque et al., 2020). Assessing niche partitioning in invaded ecosystems provides an opportunity to investigate fundamental processes in a natural setting (Garvey et al., 2020). In New Zealand, invasive mammalian predator species are subject to control, which allows the influence of individual guild members to be assessed. We recorded the spatiotemporal distribution of stoats, cats and ferrets, and their main prey (rabbits *Oryctolagus cuniculus*, rodents and birds) to test if niche partitioning was driven by prey resources and/or competition. We made the following *a priori* predictions: (1) dominant predator (cat and ferret) activity would correspond to the activity patterns of preferred prey (resource matching); (2) subordinate predators (stoats) would avoid periods of high activity by dominant predators, as opposed to matching the activity of preferred prey (safety matching); (3) site occupancy and abundance of dominant predators would correspond to the occupancy and abundance of preferred prey (resource matching), and; (4) site occupancy and abundance of the subordinate predator would be negatively correlated with those of dominant predators (safety matching). We predicted that: (5) removal of cats and ferrets would lead to increased abundance and/or occupancy of stoats, with no corresponding increase in a non‐treatment site. To test these predictions, we investigated fine‐scale spatial distributions, habitat use, prey availability, and activity patterns of sympatric invasive predators (cats, ferrets, and stoats). A pulse perturbation experiment was implemented to test whether the subordinate predator (stoat) responded to dominant predator (cat and ferret) removal. We used occupancy models (MacKenzie et al., 2002), based on trail camera detections of our study species, to determine factors that influenced distribution while accounting for imperfect detectability.

## METHODS

### Study area

This study was undertaken at two farmland sites in Hawke's Bay, New Zealand (~39° S, 176° E). Study sites were predominantly grazed pasture with a mosaic of scrub patches dominated by mānuka (*Leptospermum scoparium*) and kānuka (*Kunzea robusta*) at higher elevations (range: 220–635 m), and remnant broadleaf woodland at lower elevations (range: 120–385 m). The study sites were 15 km apart, with no recent history of predator control.

### Study species

New Zealand's three largest terrestrial carnivores – cats, ferrets, and stoats – are found across a wide range of habitats (King, 2005; King & Powell, 2007). These predators compete for resources such as space, den sites and prey, (Alterio et al., 1998; Murphy et al., 2004). Primary prey includes rodents (ship rats *Rattus rattus*, Norway rats *R. norvegicus* and mice *Mus musculus*), lagomorphs (rabbits and hares *Lepus europaeus*), birds, and invertebrates (King, 2005). In forested areas, the relative importance of birds, insects, and rodents increases, while rabbits typically predominate pasture (King, 2005; Murphy et al., 2015). Prey susceptibility to attack varies spatially and temporally depending on a predator's hunting methods; stoats hunt fossorial, terrestrial, arboreal, and aquatic prey, ferrets hunt fossorial and terrestrial prey, and cats ambush or stalk terrestrial prey (King, 2005).

Ferrets are almost exclusively nocturnal, whereas stoats may be active at any time of day (King, 2005; King & Powell, 2007; Sidorovich et al., 2008). Feral cats are adapted for nocturnal or crepuscular activity, but will modify their behavior to coincide with environmental factors such as prey activity (Fitzgerald & Karl, 1979; King, 2005).

### Camera traps and lure

**FIGURE 1:**
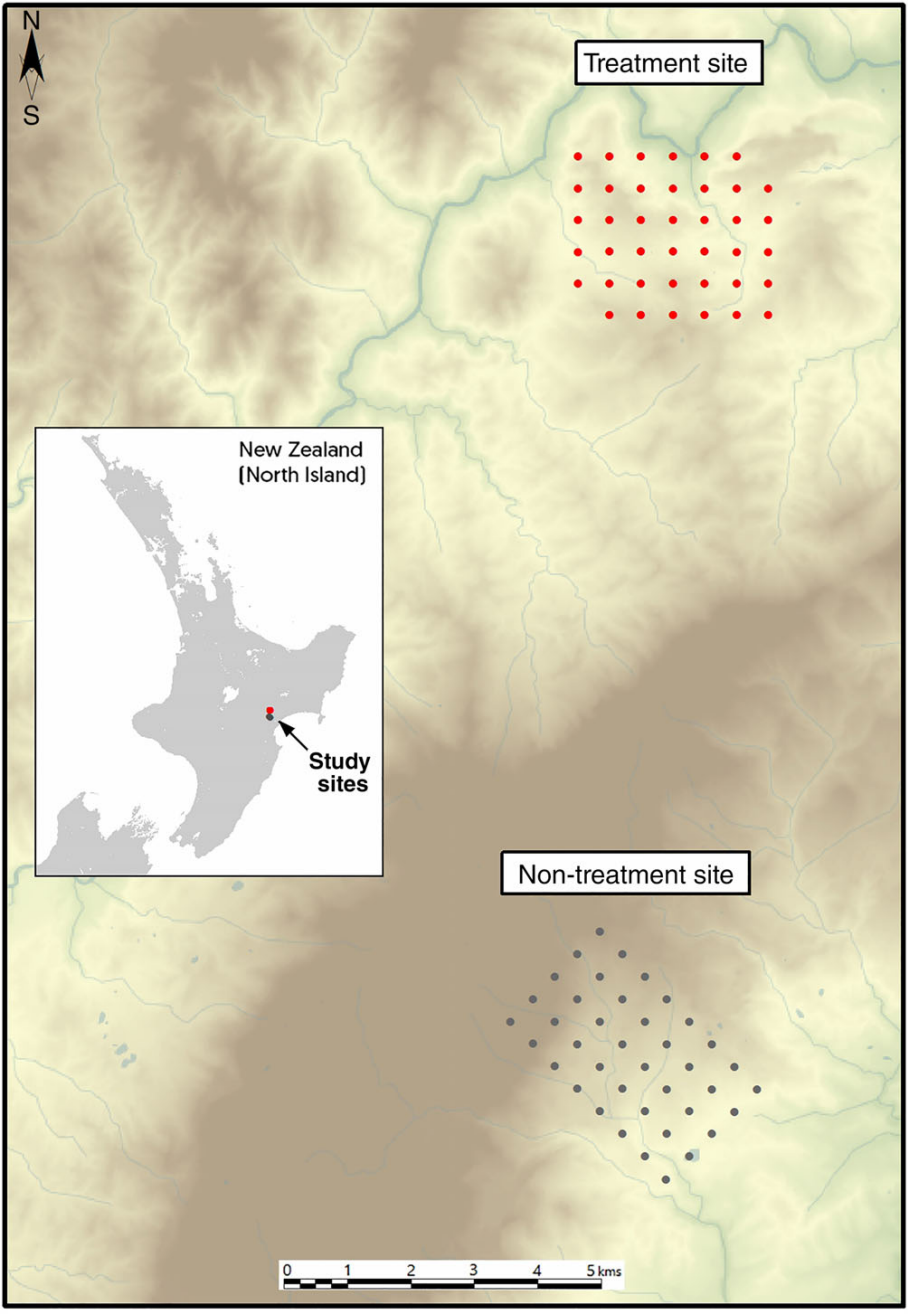
Trail camera grid at the non‐treatment (gray dots) and treatment (red dots) sites at Hawke's Bay, on the North Island of New Zealand. Eighty cameras were deployed at monitoring stations (40 at each site), with 500‐m spacing between cameras

In total, 80 (40 per study area) Reconyx PC 900 (Reconyx Inc., Holmen, Wisconsin) trail cameras were deployed, one camera at each monitoring station, with ~500‐m spacing between cameras (Figure 1). Home ranges of focal species were taken into account when deciding on camera spacing as this determines the independence of observations (Meek et al., 2012). A distance of 500 m is greater than the home range diameter for most of New Zealand's invasive mammalian predators, although feral cats in pastoral landscapes may have home ranges that exceed this camera spacing (King, 2005). However, our goal was to describe fine‐scale reciprocal distribution and habitat use and therefore our spacing was deemed optimal to balance logistical, biological, and statistical requirements. The date and time stamp recorded on each photograph were extracted using R v.3.1.1 (R Core Team, 2016), with a specially written function (Garvey et al., 2017). The time stamp on each photograph was corrected to solar time, using records of sunrise/sunsets from the study area (www.timeanddate.com/sun/new-zealand/napier), to ensure species’ activity could be matched to the diel cycle.

Cameras were fixed to a wooden post, with the base 7 cm from the ground, the approximate shoulder height of mustelids. Cameras were orientated horizontally, the most appropriate set‐up for our focal species (Nichols et al., 2017). All cameras were set to high sensitivity, three pictures per trigger and no delay between triggers. Vegetation was removed (when necessary) up to 1.5 m from the camera to allow for an unobstructed field of view and to minimize false triggers. Camera batteries and memory cards were replaced at the completion of each monitoring session.

To attract focal predators, a lure containing a combination of a social lure (ferret odor) and fresh rabbit meat (Garvey et al., 2016) was placed in a vial 1.5 m in front of the camera and secured with a wire peg. Lures were replenished at the start of each monitoring session.

### Experimental design

Camera traps were deployed in three seasons – late autumn (April), early winter (May–June) and late spring (November) 2014 – to monitor focal species at both study sites. Each monitoring session was 21 days after the day cameras were activated. Sampling grids, each covering 7 km^2^, were established in a treatment (predator removal) site and a non‐treatment site (Figure 1). Habitat type (open pasture or scrub) was assessed by visual inspection of the dominant vegetation within a 20‐m radius of each camera trap and subsequently coded as a binary variable. A 20‐m radius was selected to correspond with the approximate field of view of the camera and to ensure habitat variables were assessed based on the dominant vegetation type at a monitoring stations.

Predator occupancy and detection probabilities are often a function of site characteristics, such as habitat variables (MacKenzie et al., 2002). We predicted that ferrets would select against scrub habitat, where poor climbing abilities limit access to prey, but would select for open pasture where primary prey (lagomorphs) were more abundant. We had no *a priori* prediction for the influence of habitat on cats and stoats, which are generalist predators. However, interference competition tends to be stronger in homogeneous habitats (Finke & Denno, 2002) and this may limit stoat distribution in open pasture.

A before–after control–impact (BACI) design was implemented to test whether stoats responded to dominant predator (cat and ferret) removal. After 3 weeks of pretreatment monitoring in May 2014, feral cats and ferrets were removed by professional trappers during a pulse perturbation on the treatment site, while the non‐treatment site remained unperturbed.

An additional camera‐trap trial was conducted at the non‐treatment site in summer (January–February) 2014 as part of a separate project (Garvey et al., 2017). Both studies implemented the same study design to monitor stoats, although cameras were placed at scrub/pasture margins in the earlier experiment. Information from this earlier summer deployment was used to supplement data on stoat temporal activity.

### Data recording

We considered each camera trap to be an independent sampling unit and analyzed data based on observations of both predators and prey. We calculated the following variables for analysis: (1) independent observations, and (2) site use. To avoid autocorrelation, we considered independent observations to be photographs of the same species on the same camera more than 30 min apart, except when individuals could be reliably distinguished (Linkie & Ridout, 2011). The 30‐min timeframe was selected after plotting histograms of time elapsed between consecutive photographs for each species (Brook et al., 2012). Site use is a binary response variable with 1 indicating a species was detected and 0 indicating non‐detection. Predator detections may be non‐independent when surveying mobile animals, as the part of a home range used in a 21‐day monitoring session may encompass more than one camera (Mackenzie, 2006). We therefore relaxed the assumption of closure to interpret detection probabilities as site use rather than site occupancy, which we defined as the proportion of sampled sites used by a species (MacKenzie et al., 2017; Neilson et al., 2018). We also recorded any characteristic predator behaviors apparent in the photographs such as capture of prey or response to the scent lure (Garvey et al., 2017).

### Activity

Cameras recorded the time and date of each photograph, providing temporal data on animals’ activity. We characterized activity patterns for each predator separately with kernel density estimates; these are non‐parametric representations of the probability density function for a continuous random variable. Species’ activity was classified into four categories: diurnal (between 1 h after sunrise and 1 h before sunset); nocturnal (between 1 h after sunset and 1 h before sunrise); cathemeral (throughout the 24 h period) and crepuscular (up to1 h before and after sunrise and sunset) (Linkie & Ridout, 2011). We compared activity patterns to assess the potential for interactions between pairs of species by calculating the coefficient of overlap (Δ), which varies between 0 (no overlap) and 1 (complete overlap). We quantified the coefficient of overlap as low (0.0–0.33), moderate (0.34–0.66), or high (0.67–1.0). We had sufficient cat and ferret observations across all seasons to use the estimator for large sample sizes (Δ_4_) but used the lowest threshold (∆_1_) for stoats, based on the number of observations (please refer to Ridout & Linkie, 2009). Confidence intervals were obtained by bootstrapping samples 10,000 times. Statistical analyses were implemented using the *overlap* package (Ridout & Linkie, 2009) in the software R (R Development Core Team, 2016).

Before pooling data on stoat temporal activity from the two camera‐trap trials, we calculated the coefficient of overlap (Δ_1_) to ensure that activity patterns did not differ between trials at the non‐treatment site. Following the methods of Linkie and Ridout (2011), a Δ_1_ value of 0.95 indicated that activity data matched substantially (Δ ≥ 0.90) and could therefore be combined. Prior to combining data, we confirmed, using the coefficient of overlap (Δ_1_), that there were no significant differences in the activity patterns of focal predators among sites and between seasons (Linkie & Ridout, 2011). Prey species were grouped when there were no significant differences in activity patterns: rats and mice were grouped into a category “rodents” and all bird species were placed in one group.

### Predator distribution and contributing factors

Monitoring sessions were divided into seven intervals of 3 days. We ran single‐season occupancy analyses within monitoring sessions where predator detections were sufficient to investigate factors that influenced distribution on a landscape scale. These analyses were conducted for cats and ferrets at the treatment site in the preperturbation period, and for stoats and cats at the non‐treatment site in all monitoring sessions. Due to low levels of detections, naïve site use (proportion of sampled units where a predator was detected) was calculated in other seasons.

To assess mesopredator occupancy as a function of dominant predators, we ran a single‐season stoat model. Apex predator (cat/ferret) and prey (rodent/lagomorph/bird) were included as linear covariates in the psi (ψ) conditional model, where psi refers to the probability that a site is occupied by a species of interest (MacKenzie et al., 2017). A species interaction model was also created to assess the spatial variations in site use patterns between cats and ferrets (Richmond et al., 2010). Cats were selected as the dominant species in the model, which we predicted based on their larger size and the propensity of felids to engage in interspecific killing (Donadio & Buskirk, 2006; Palomares & Caro, 1999). An odds ratio was calculated on the probability of occupancy and detection to quantify the level of dependence between apex predators, where values >1 suggest that a species’ site use is greater than expected under independence (v = 1) (MacKenzie et al., 2017). All occupancy analyses were undertaken in R and the program PRESENCE 9.0 (Hines, 2006).

Models were created to investigate the influence of prey and habitat covariates on patterns of predator occupancy and detections. Candidate single‐season models for ferrets and cats were assessed based on the global model: *logit* (*ψi*) = *ψ* (*Rb* + *Rd* + *B* + *S* + *P*), *p*(*Rb* + *Rd* + *B* + *S* + *P*), where prey covariates relate to rabbits (*Rb*), rodents (*Rd*), and birds (*B*), habitat covariates are scrub (*S*) or pasture (*P*), *p* is detection and *ψ* is occupancy. Covariates were selected because they have been shown to influence the distribution of our predators or represent landscape habitat attributes that we predicted may influence predator occurrence or detectability. Prey were included as linear covariates based on the conditional occupancy estimates (*ψ*‐cond) of prey occurrence for each site and habitat covariates were coded as binary variables where a monitoring station was either located on scrub or pasture. The scent lure was not included in our hierarchical models as every site received the same lure treatment and a previous study demonstrated the attractiveness of the scent lure for our target species (Garvey et al., 2017). For the stoat single‐season model, an additional binary variable cat (*C*) was included based on the observed presence (1) or absence (0) of this predator at monitoring stations. Following MacKenzie et al. (2017), we first modeled detection probabilities as a function of covariates, while holding the full set of occupancy covariates constant (rabbit, rat, bird, pasture, scrub), which allowed inferences about predator detection efficiency with as few constraints as possible. We then specified different combinations of occupancy against the top detection probability model for the full set of candidate occupancy models. A constant‐detection model was also included, as this model assumes occupancy is not affected by imperfect detection and therefore holds detection constant across surveys and sites. Covariates that influence detection probabilities are included in the results tables and otherwise the null model is presented. We calculated Pearson's correlation coefficients to ensure modeled variables were not highly correlated (>0.7). Parameter estimates and covariate beta coefficients (b) are presented ± standard error (Mackenzie, 2006). We ran a goodness‐of‐fit test on the global model to ensure that the fitted model adequately described the observed data and we selected quasi‐AIC_c_ (QAIC_c_) where we identified overdispersion (MacKenzie et al., 2017). For model comparison, we used AIC with small sample correction (AIC_c_), and Akaike weights (w) were used to determine the best supported models for each species (Burnham & Anderson, 2002). Models that were within two AIC_c_ units (*∆*AIC_c_) of the top ranked model were considered as final candidate models. To be consistent with our AIC‐based approach to model assessment, we present beta coefficients with ~85% confidence intervals (CIs) based on unconditional, VIF‐inflated SEs (Arnold, 2010). Differences in predator detection/non‐detections at a site and whether behavioral responses (i.e. stoat contact with the lure) varied by time of day were assessed using Fisher's exact test.

## RESULTS

A trapping effort of 4405 trap days generated 769,408 photographs, of which 19,120 were of the focal mammalian predators and prey. There were an additional 26,212 photographs of 27 bird species (13 native, 14 introduced) (Appendix S1: Table S1). For the focal mammalian prey, there were 421 and 570 independent observations of rabbits and rodents, respectively. More than 90% of identified *Rattus* spp. were ship rats, with the remainder Norway rats. We recorded 264 independent observations of four carnivores: feral cats 168 (64%), ferrets 72 (27%), stoats 23 (8%), and a weasel 1 (<1%). There were an additional 35 stoat observations in the preliminary camera‐trap trial. Livestock (cattle, sheep, and deer) accounted for the majority of photographs at both sites. Cameras were deactivated on 11 of 240 sampling occasions due to livestock disturbance or camera malfunctions. The number of active camera traps, trap days, and photographs are summarized in Table 1.

**TABLE 1:** Summary of camera trapping and numbers of independent observations of predators (cats, ferrets, stoats) and primary prey (rabbits, rodents)

Study site	Trial period	Camera traps	Camera‐trap days	Predator detections	Prey detections
Non‐treatment	April	38	797	58	83
	May–June	38	797	63	116
	November	38	722	47	284
	Non‐treatment total		2316	168	483
Treatment	April	40	822	78	211
	May–June	37	648	6	154
	November	38	619	11	143
	Treatment total		2089	95	508

### Temporal activity patterns

**FIGURE 2:**
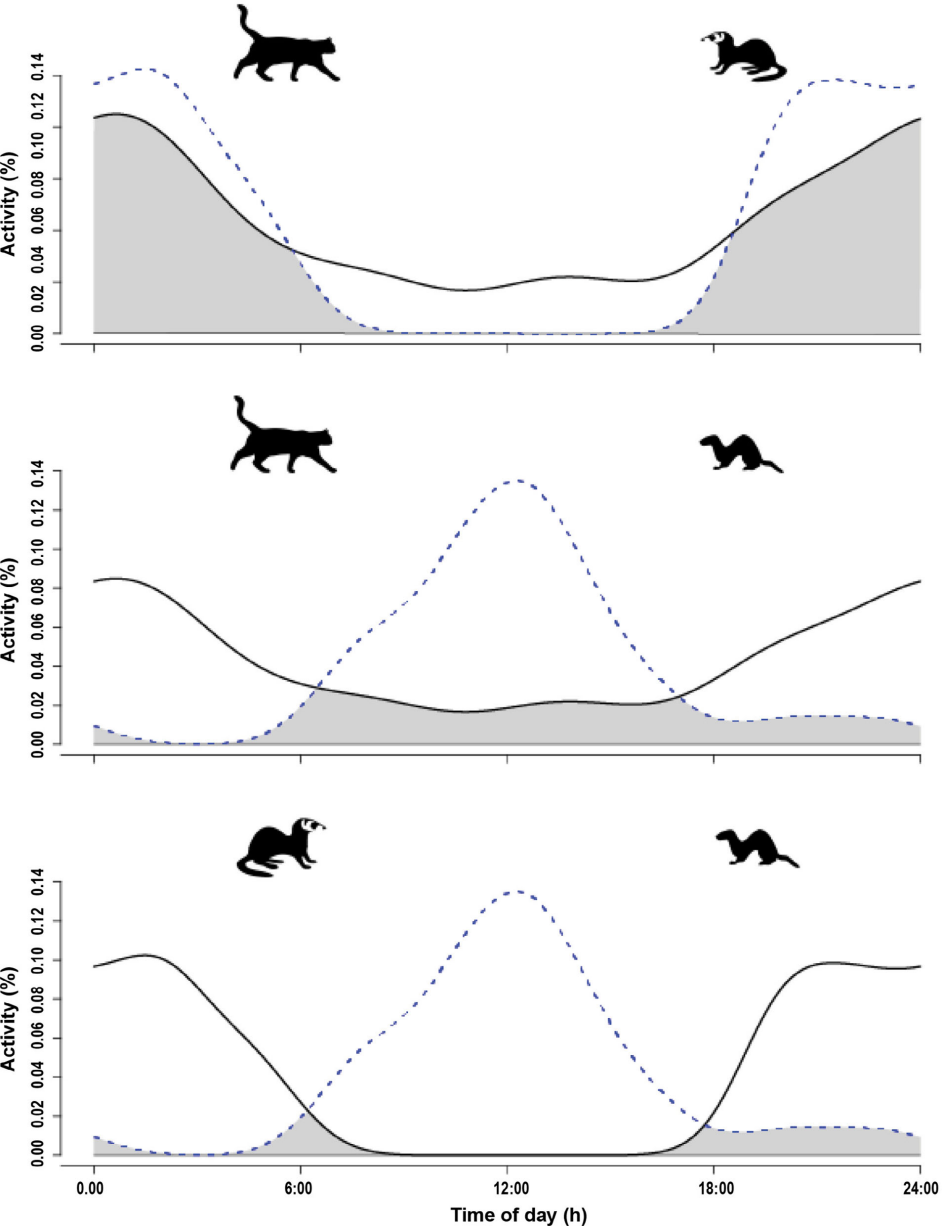
Pairwise comparison (from top: cat vs. ferret, cat vs. stoat, ferret vs. stoat) of the daily activity of three invasive predators. The larger predator in the pairing (left symbol) is represented by a solid line and the smaller predator (right symbol) by a dashed line. Overlapping periods of activity are shaded as gray. Species' activity patterns are displayed over the 24 h dial period, which we re‐scaled to equal periods of daylight (6:00 ‐ 18:00) and night (18:00 ‐ 6:00)

Activity patterns varied within the predator guild and among their prey. Cats were cathemeral, with activity lowest during the day and reaching a peak just after midnight. Ferrets were exclusively nocturnal, with all 72 observations occurring between dusk and dawn. Activity patterns for the two dominant predators overlapped substantially (Table 2, Figure 2).

**TABLE 2:** Overlap in temporal activity among predators and prey

	Stoat (Δ_1_)	Cat (Δ_4_)	Ferret (Δ_4_)
Stoat	–	–	–
Cat	0.38 (0.19–0.51)	–	–
Ferret	0.13 (0.05–0.21)	0.74 (0.62–0.90)	–
Rabbit	0.55 (0.44–0.65)	0.56 (0.65–0.78)	0.33 (0.26–0.42)
Rodent	0.12 (0.05–0.20)	0.72 (0.61–0.84)	0.88 (0.82–0.94)
Bird	0.80 (0.72–0.88)	0.36 (0.24–0.53)	0.09 (0.05–0.13)

*Note*: Activity patterns were compared with assessing the potential for interactions between pairs of species by calculating the coefficient of overlap (Δ). Cats and ferrets had sufficient observations to use the estimator for large sample sizes (Δ_4_), while the lower threshold (*∆*_1_) was used for stoats. Bootstrapped 95% confidence intervals are included in parentheses.

In contrast, stoats were almost entirely diurnal, with a 0.91 kernel probability of being active during the day, and a third of activity taking place 1 h either side of noon. There were nocturnal observations of stoats on five (9%) occasions during the summer months (January–February). Stoats displayed minimal overlap with the two dominant predators (Table 2, Figure 2); stoat activity corresponded closely with the period of low cat activity, while stoats and ferrets had opposite activity patterns (Table 2, Figure 2). Rodents were entirely nocturnal, except for a small number of mouse sightings (<2%) during daylight hours. Birds were diurnal, appearing immediately after first light, with one observation (morepork *Ninox novaeseelandiae)* at night.

### Predator overlap with prey

**FIGURE 3:**
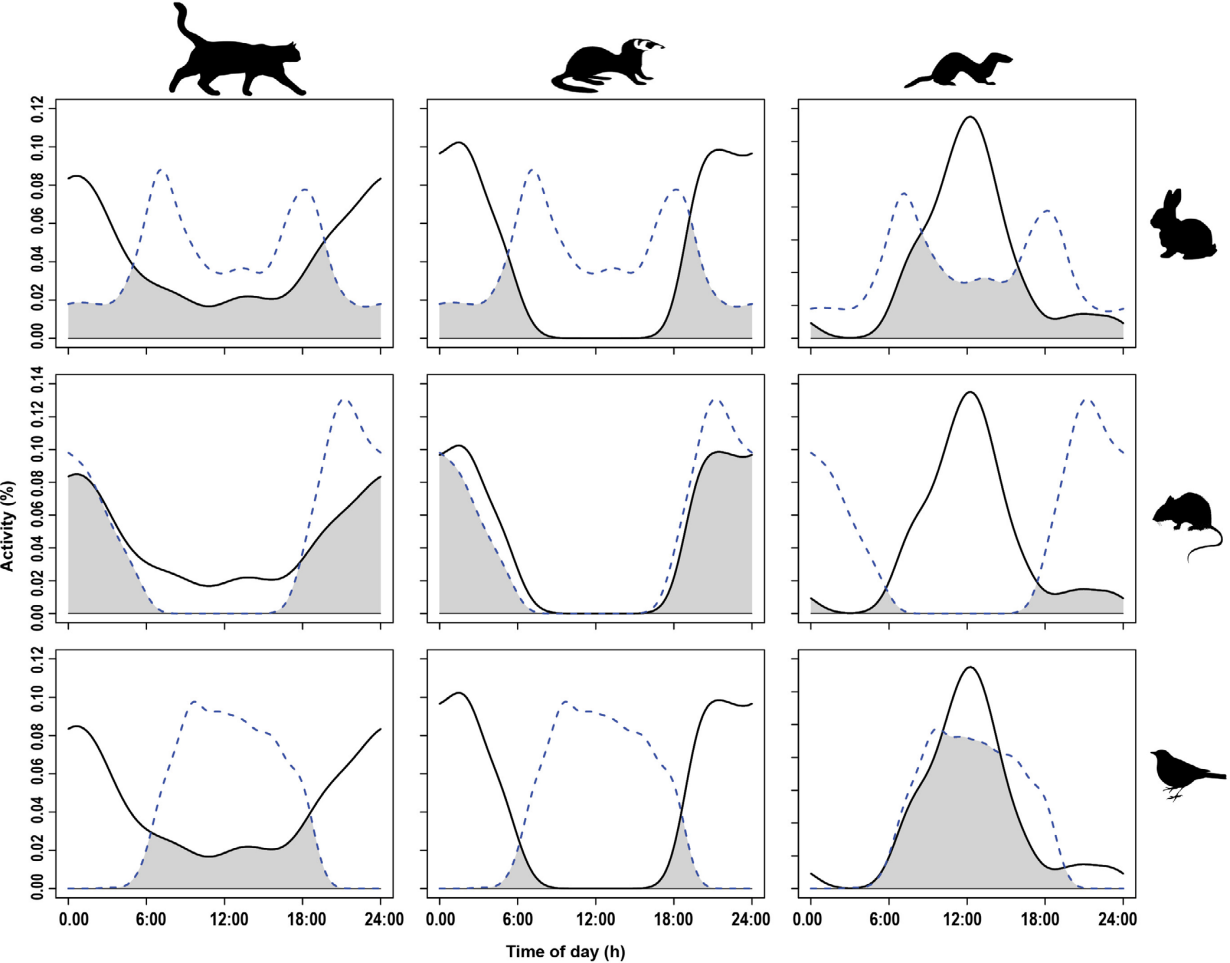
Predator (cat, ferret, or stoat; solid lines) overlap in activity with the activity of primary prey species (rabbits, rodents, or birds; dashed line). Overlapping periods of activity are shaded as gray

Cathemeral activity of cats corresponded broadly with all three prey groups (Table 2, Figure 3). Ferrets displayed very high overlap with rodents, but comparatively low overlap with rabbits and birds (Table 2). Ferrets were recorded at only four camera traps in the non‐treatment site, with rabbits recorded at three of these. Stoat diurnal activity resulted in minimal overlap with rodents (Table 2, Figure 3). Stoats displayed high overlap with rabbits, with evidence of a slight shift in activity toward peak rabbit activity at dawn (Table 2, Figure 3). Stoats exhibited the greatest overlap with birds (Table 2, Figure 3).

### Predator interactions

The composition of the predator guild differed between sites, although cats were the most frequently detected predator at both locations (Appendix S1: Table S2). There was no evidence of competitive exclusion among top predators (Table 3) as the probability of a ferret occupying a site was greater when cats were present (*ψ* ^B/A^ = 0.50, SE = 0.80) than absent (*ψ* ^B/a^ = 0.31, SE = 0.63). Ferrets were more than twice as likely to occupy (odds ratio; *v* = 2.31) and be detected (odds ratio; *v* = 2.0) at a site where a cat was recorded. Cats and ferrets co‐occurred at 36% of camera traps within a monitoring period, with sequential sightings less than 5 h apart on three occasions.

**TABLE 3:** Summary of cat and ferret interaction models at the treatment site preperturbation

Parameters	Description	Probability	Coefficients (beta)	SE
*ψ*A	Probability of cat occupancy	0.51	0.02	0.51
*ψ*BA	Probability of ferret occupancy, given cats present	0.50	0.02	0.80
*ψ*Ba	Probability of ferret occupancy, given cats absent	0.31	0.82	0.63
*p*A	Probability of detecting a cat given site occupied	0.25	1.09	0.59
*p*B	Probability of detecting a ferret given site occupied	0.66	0.65	0.53
*r*A	Probability of detecting a cat, given both species present	0.18	1.51	0.52
*r*BA	Probability of detecting a ferret, given both species present, and a cat is detected during the survey	0.40	0.42	0.90
*r*Ba	Probability of detecting a ferret, given both species present, and a cat is not detected during the survey	0.25	1.11	0.71

A single‐season occupancy model revealed the importance of apex predators on stoat site use (Table 4). Cats (b = −1.65, SE = 1.14; CI −0.01 to −3.29) had the greatest influence on stoat distribution, appearing as a negative covariate in every model that had substantial support (Table 4). The model with the ferret covariate did not converge, as there was only a single ferret detection at a site where stoats were not detected. In fact, both mustelids never occurred at the same monitoring station within a recording session. Post‐perturbation, stoats were detected at two stations previously occupied by apex predators, and all three predators were never recorded at a monitoring station within the same sampling period.

**TABLE 4:** Summary of top models for predators (cat, ferret, and stoat) that received substantial support (*Δ*AIC_c_ < 2)

Model (QAIC_c_ < 2)	QAIC_c_	ΔQAIC_c_	QAIC_c_ wgt	Likelihood	Number of Parameters
Cat					
Both sites					
psi(R),p(L)	259.02	0	0.23	1	3
psi(.),p(.)	259.06	0.04	0.22	0.98	2
psi(P),p(L)	260.63	1.61	0.10	0.45	3
psi(B),p(L)	260.77	1.75	0.09	0.42	3
psi(L),p(L)	260.83	1.81	0.09	0.40	3
psi(P + S),p(L)	260.87	1.85	0.09	0.40	4
psi(R + B),p(L)	260.96	1.94	0.09	0.38	4
psi(R + L),p(L)	260.97	1.95	0.09	0.38	4

Model (AIC_c_ < 2)	AIC_c_	ΔAIC_c_	AIC_c_ wgt	Likelihood	No. Partitioning
Control site					
psi(P),p(B)	150.04	0	0.24	1	3
psi(R),p(B)	150.09	0.05	0.24	0.98	2
psi(.),p(.)	150.72	0.68	0.17	0.71	2
psi(S + P),p(B)	151.12	1.08	0.14	0.58	4
psi(R + P),p(B)	151.62	1.58	0.11	0.45	4
psi(S),p(B)	151.77	1.73	0.10	0.42	3
Removal site					
psi(L),p(R)	107.12	0	0.38	1	3
psi(R + L),p(R)	107.76	0.64	0.28	0.73	4
psi(L + B),p(R)	108.5	1.38	0.19	0.50	4
psi(R + L + B),p(R)	108.98	1.86	0.15	0.39	5
psi(.),p(.)	109.78	3.33	0.04	0.19	2
Ferret					
Removal site					
psi(L + S),p(.)	117.01	0	0.83	1	3
psi(S),p(.)	121.51	4.5	0.09	0.10	2
psi(L),p(.)	121.65	4.64	0.08	0.10	2
psi(.),p(.)	129.03	7.54	0.04	0.08	2
Stoat					
Control site					
psi(C + L + B,p(C)	134.44	0	0.45	1	5
psi(C + L),p(C)	134.83	0.39	0.37	0.82	4
psi(C),p(C)	136.31	1.87	0.18	0.39	3
psi(.),p(.)	138.08	8.12	0.01	0.02	2

*Note*: Models for cats and ferrets evaluate the impacts of covariates—prey (rat, lagomorph, bird) and habitat (pasture or scrub)—on predator site use and probability of detection. A stoat single‐season model evaluates the influence of an apex predators (cat) and prey (rat/lagomorph/bird) on stoat site use and probability of detection at the non‐treatment site, using the psi (*ψ*) conditional model of these covariates. Models are presented in ascending order based on AIC_c_ values. ΔQAIC_c_ is the difference in the QAIC_c_ value of each model compared with the QAIC^c^ for the top model and QAIC_c_ wgt is the Akaike weight for each model. Covariates are abbreviated as follows: predator (cat, C; ferret, F), prey (rodent, R; lagomorph, L; bird, B) and habitat (pasture, P; scrub/forest, S).

### Spatial distributions

Prey resources varied considerably between study sites (Appendix S1: Table S3). Rats were the most common prey at the treatment site, with naïve site use of 45%, and there were 17 times more independent observations of rodents (265 vs. 16) at the treatment site preperturbation.

This trend was reversed for other prey; there were four times more independent observations of rabbits at the non‐treatment area (132 vs. 32), with naïve site use of 49%. There were approximately four‐fold more independent observations of birds at the non‐treatment site (430 vs. 118). Introduced bird species, which made up more than 80% of all independent observations, were primarily in pasture (10 of 14 bird species recorded in this habitat were introduced), while native species were more common in scrub/forest patches (9 of 13 species recorded were native) (Appendix S1: Table S1).

#### Association of prey and habitat types with predator distribution

Predator observations were driven by prey and habitat type, particularly for apex predators (Table 4). For cat models, AIC_c_ was selected based on the sample size and where both sites were analyzed together, QAIC_c_ was selected as overdispersion was identified by the goodness‐of‐fit test. When both study sites were analyzed together, rodents appeared as a positive covariate in three of the eight top single‐season models for cats. Cats selected against the habitat variable pasture (b = −0.77, SE = 0.30; CI: −0.34 to −1.20). At the treatment site, prey were associated with cat site use, appearing in every model; the top four models all included the rabbit covariate, which was selected against (b = −40.38, SE = 13.16; CI: −38.52 to −42.24). Cat probability of detection was positively associated with rodents (b = 0.75, SE = 0.31; CI: 0.30 to 1.20), the most common prey at the site. Habitat did not influence detection probabilities as neither variable appeared in the top supported models.

The top model for ferrets identified just two important covariates; ferrets were positively associated with rabbits (b = 1.85, SE = 1.05; CI: 3.37 to 0.35) and pasture (b = 2.55, SE = 1.22; CI: 4.30 to 0.79). The top model with both these covariates was the only model supported with AIC_c_ <2. Ferret probability of detection was not associated with prey covariates, with the null model having greatest support.

The stoat model revealed that stoat site use was negatively associated with the lagomorph and bird covariates, which had a combined model weighting of 0.55, while the cat covariate accounting for the remaining model weight.

Of the five camera traps where stoats were detected at 6 months post‐control, both cats and ferrets had been previously recorded at one camera trap and cats had been found at two others prior to the pulse perturbation. Rodents were recorded at all five of the camera traps where stoats were detected, birds at four, and rabbits at two.

### Dominant predator removal

**FIGURE 4:**
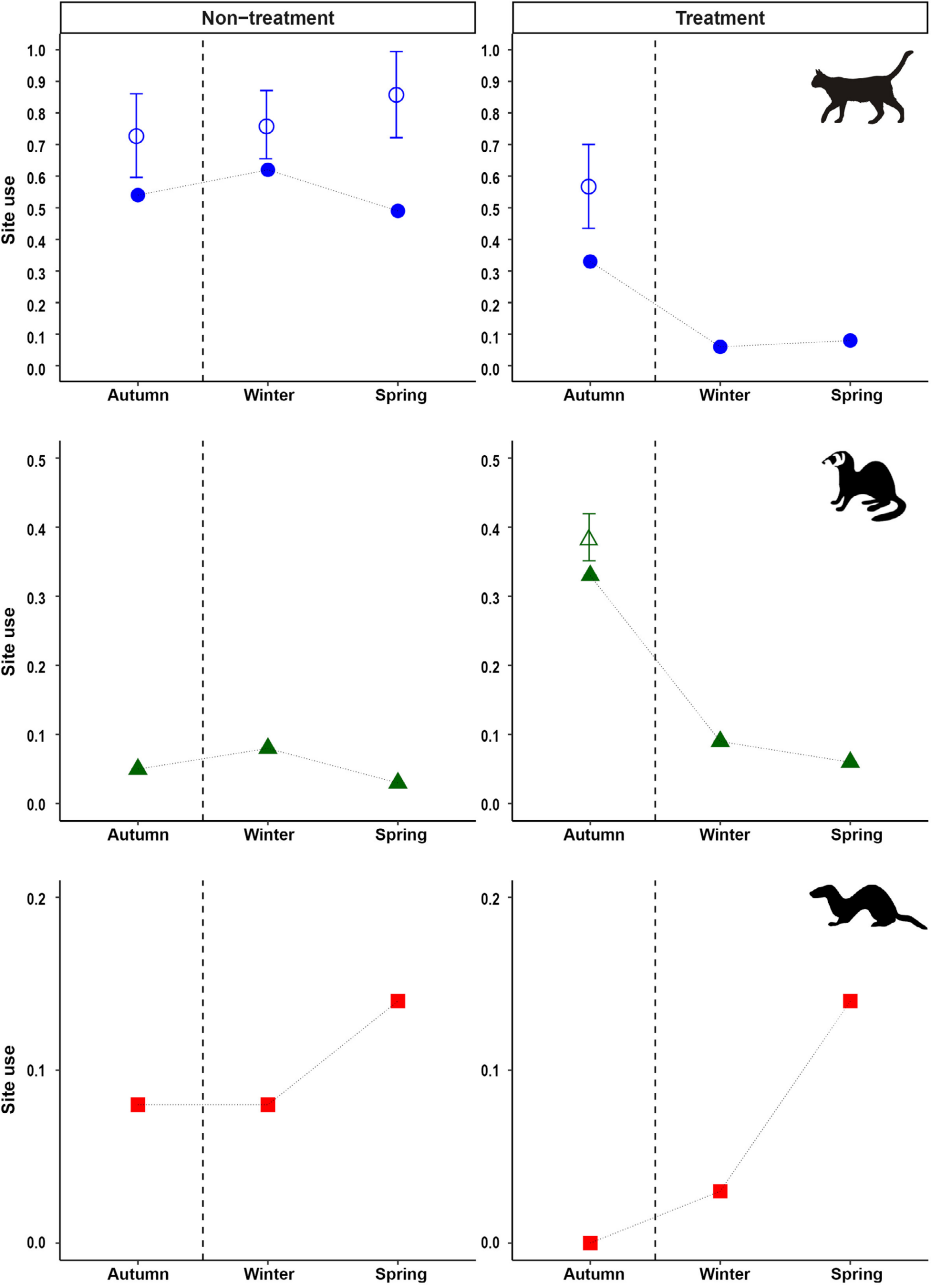
Site use estimates (±95% CI) and naïve site use (no error bars) for cats (●), ferrets (▲), and stoats (■) at non‐treatment and treatment (predator removal) sites

Specialist trappers removed 17 cats and 18 ferrets from the treatment site during the 3‐week pulse perturbation. Site use models showed a significant decrease in cats (*p* = 0.0004) and ferrets (*p* = 0.0021) following predator removal, representing a fall of 82% and 76%, respectively (Figure 4; Appendix S1: Table S2). In contrast, site use estimates at the non‐treatment site showed a slight increase for cats and a slight decrease for ferrets over the same period (Figure 4; Appendix S1: Table S2).

Naïve site use was used to assess stoat distribution as low numbers of detections precluded the use of occupancy estimates. No stoats were recorded at the treatment area prior to the removal of cats and ferrets, but stoats were observed at 14% of monitoring stations 6 months post‐perturbation (Figure 4; Appendix S1: Table S2), which was a significant increase in stoat detections over the period (Fisher's exact test, two‐tailed: *p* = 0.0218). A third of the sites where stoats were detected had been previously occupied by both apex predators. At the non‐treatment site, stoat observations were relatively constant over the recording periods (Figure 4; Appendix S1: Table S2), with a non‐significant increase in detections (Fisher's exact test, two‐tailed: *p* = 0.71).

#### Additional observations

There were 13 instances where a stoat approached and contacted the lure vial. There was a significant difference between behaviors depending on the time of day (Fisher's exact test, two‐tailed: *p* = 0.0033, *n* = 13), as stoats approached ferret odor during daylight but never at night. Predators were recorded with prey on two occasions: a cat was photographed with a rodent at the treatment site in May and a stoat dragged a dead rabbit to the lure at the non‐treatment site during November (Appendix S1: Figures S1, S2).

## DISCUSSION

Our results support the existence of niche partitioning among competing invasive predators, driven substantially by the availability of abundant invasive prey. Dominant predators displayed resource matching by aligning their temporal activity and spatial distribution to maximize access to primary prey. Cats and ferrets exhibited high niche overlap as both species were predominantly nocturnal and distributed based on primary prey resources. However, niche partitioning was achieved as they targeted different prey (rabbit vs rodents) and selected different habitat types. Surprisingly, given the ubiquity of interference competition among top order predators, there was no evidence of competitive exclusion between the two apex predators; ferrets were more than twice as likely to occupy and be detected at a station where a cat was recorded. Ferrets are tenacious, with a bite force equivalent to the much larger domestic dog (*Canis familiaris*) (Dessem & Druzinsky, 1992), so, despite their larger size, cats may avoid agonistic interactions with ferrets due to the risk of injury. As both predators can eat similar prey, different hunting strategies may reduce niche overlap and facilitate coexistence.

Stoats coexist as the subordinate species of the predatory guild by reducing spatial and temporal exposure to dominant predators. This avoidance strategy would be likely to have associated fitness constraints, as it would lead to reduced overlap with primary prey. We found strong evidence of temporal partitioning, supporting the niche‐complementarity hypothesis, which predicts that high overlap in one dimension will be compensated for by low overlap in another (Loreau et al., 2002). Exploitation competition is unlikely to be the limiting factor, as rabbits and rodents were locally abundant and stoat reliance on diurnal prey will reduce resource overlap with dominant predators. Stoat ability to access arboreal and burrowing prey may facilitate vertical niche partitioning, particularly in scrub areas where ferrets are absent. In scrub habitat, interference competition with cats will lead to stoats persisting at lower densities, as every stoat site use model contained cats as a negative covariate. The combined competitive force of cats and ferrets may suppress stoats from farmland, but this might depend on the proportion of scrub patches versus pasture. This may explain why stoats were only detected at the treatment site following the pulse perturbation, although an overall low number of detections made it difficult to draw firm conclusions.

### Temporal activity

Dominant predators displayed activity patterns that corresponded to the availability of prey resources. Cats were predominantly active at night, with temporal patterns closely tracking that of rodents, but were also moderately active throughout the day, resulting in cats having the highest overlap with all three prey types. Cats are cryptic predators, so prey vulnerability should closely correspond to prey activity. Ferrets were exclusively nocturnal, which initially seemed counterintuitive as this minimized temporal overlap with their primary food resource, rabbits (King, 2005). However, ferret spatial distribution was highly correlated with that of rabbits, suggesting a high reliance on this species. Ferrets are predominantly subterranean hunters and appear to match their temporal activity to prey vulnerability, when rabbits are least active above ground. Ferret nocturnal activity overlapped with rodent activity, which can be an important food resource for juveniles and females, but the lack of spatial overlap suggests that rodents were not an important resource. Activity patterns of stoats contrasted with the temporal patterns of dominant predators, leading to a reduced overlap with preferential prey (safety matching). Stoats were almost exclusively diurnal, with camera records peaking at midday, corresponding to the low point of activity for dominant predators. Cathemeral activity of cats precluded the complete avoidance by stoats, yet temporal overlap between the species was only 38%. Stoats avoided ferrets by displaying opposite diel activity cycles, considerably reducing the risk of encounter.

Stoats displayed low temporal overlap with rodents, which is surprising as rats and/or mice are usually the main dietary component, particularly in forested areas (Jones et al., 2011). Rats are vulnerable to stoat predation when in burrows, but ship rats, the most frequently recorded rodent at our study sites, usually occupy arboreal nests when inactive (King, 2005). Stoats displayed the greatest overlap with rabbits of any mammalian prey. Stoats frequently hunt rabbits above ground (King & Powell, 2007), requiring concurrent activity patterns, yet stoats failed to align with the crepuscular peak of rabbit activity.

### Spatial partitioning and resource matching

Predators did not display spatial segregation at a landscape scale (i.e., entire study area), but did so at the home range scale (i.e., monitoring stations). Cats and ferrets were active concurrently but selected for different habitats. Prior to the perturbation, the apparent absence of stoats suggests they are prone to competitive exclusion in pasture‐dominated ecosystems where spatial avoidance is difficult. Mustelids never co‐occurred at a camera within a recording period, but stoats co‐occurred with cats on six occasions. Nocturnal activity patterns displayed by cats at these camera traps contrasted with the diurnal activity of stoats, reducing the risk of an encounter. On occasions when stoats were nocturnal they did not approach the lure at a camera trap, which contrasted with behaviors recorded in daylight hours when the absence of dominant predators could be confirmed visually.

The results generally supported our prediction of resource matching, as dominant predators were positively associated with preferred prey across monitoring stations. Cat detection probabilities were driven by rodents and this conclusion was supported by observations of concurrent nocturnal activity. Unexpectedly, cats avoided pasture with rabbits, particularly at the treatment site, where rabbits appeared as a negative covariate in all models. However, ferret distribution at the treatment site was primarily driven by rabbits and differentiation along the prey resource dimension may facilitate coexistence of ferrets and cats. In support of our prediction, stoat distribution corresponded with safety matching, being absent prior to the pulse perturbation, but subsequently recorded after removal of dominant predators.

### Mesopredator release

Stoat population densities are frequently low in New Zealand and conclusive evidence for trophic cascades is therefore elusive. In support of our mesopredator release prediction, stoats were only detected after dominant predator removal and, 6 months later, stoats had changed from being undetected to the most common predator at the treatment site. An alternative explanation is that seasonal variations in behavior, such as changes in activity patterns in response to prey availability (e.g., fledgling birds), may explain increased detections of stoats at the treatment site in the final recording period. However, there was no evidence that predator site use changed at the non‐treatment site and other authors have reported no change in predator activity over similar time periods (Alterio et al., 1998). Although the paucity of data prevented us from drawing definitive conclusions, our results are consistent with the mesopredator release hypothesis when increases in stoat detections are considered in the light of spatiotemporal partitioning, captive experiments involving these predators (Garvey et al., 2015), evidence for interspecific killing (e.g., Wodzicki, 1950), and avoidance of top predators (Keedwell & Brown, 2001; Pierce, 1987).

### Invasive species management

The New Zealand government recently adopted an ambitious goal to eradicate stoats, ship rats and possums (*Trichosurus vulpecula*) from the entire country by 2050 (Russell et al., 2015). Selectively removing a portion of the invasive predator guild from a complex network of interacting species requires an understanding of changing spatial and temporal relationships to prevent undesirable outcomes (Doherty et al., 2015). Our study revealed the complex dynamics among top order predators, a trophic level that has a disproportionate influence on ecosystem structure. We believe that a holistic approach to invasive species management is important, but slow recolonization by apex predators following the perturbation, and a concurrent increase in stoat detections, suggests that interspecific competition warrants consideration in any management plan. Controlling all New Zealand's invasive mammals without unexpected and potentially adverse impacts remains a serious challenge.

Where the entire suite of invasive predators cannot be managed simultaneously, our research supports the proposal to initially eradicate stoats, as the removal of apex predators may result in mesopredator release (Rayner et al., 2007). However, the removal of stoats, or any introduced predator, from a system dominated by invasive species could lead to the release of invasive prey. While prey release would be anticipated, the magnitude and consequences of this risk is currently unknown, and post‐removal monitoring would be necessary to understand the consequences for other species (Ruscoe et al., 2011). Invasive prey strongly influenced the distribution of apex predators, highlighting an important consideration for New Zealand's predator‐free goal. Cat site occupancy was driven by rodents, primarily ship rats, which are a species targeted for eradication by 2050. Removal of ship rats may lead to increased predation pressure on native birds, at least in the short term. A stratified control program could be considered to reduce the risk of prey switching.

Our study provides strong evidence that niche partitioning facilitates invasive predator coexistence. Collectively, the predator guild was active throughout the diel cycle and across habitat types, maximizing exploitation of the fundamental niche. Alterio and Moller (1997) have suggested that stoats pose the main predation threat to diurnal species and our results support this conclusion. Conversely, by suppressing stoats, cats may reduce the pressure on native species that are particularly susceptible to stoat predation (e.g. cavity nesting birds), although this may not compensate for the direct negative impacts that cats inflict on native species.

This research highlights the difficulties in managing invaded ecosystems, where complex interactions between native and introduced biota occur at landscape scales, and well intentioned perturbations can have detrimental impacts. Understanding the interactions among species in invaded systems will inform the timing, extent, and risks of management intervention. Future research could quantify how cascading effects of trophic interactions will influence the predation risk of native species.

## CONFLICT OF INTEREST

The authors declare no conflict of interest.

## AUTHOR CONTRIBUTIONS

Patrick M. Garvey, Alistair S. Glen, Mick N. Clout and Roger P. Pech wrote the main manuscript text and Patrick M. Garvey prepared figures. Patrick M. Garvey, Alistair S. Glen and Margaret Nichols carried out fieldwork. Patrick M. Garvey and Margaret Nichols reviewed camera trap images. All authors reviewed the manuscript.

## Supporting information


Appendix S1

## Data Availability

Data are available in New Zealand's Biological Heritage Data Repository at https://doi.org/10.34721/0cxt-v270.
