# Supplementary material for: Niche partitioning in a guild of invasive mammalian predators
Source: Ecol Appl. 2022 Mar 24;32(4):e2566. doi: 10.1002/eap.2566 (PMC9285952; doi:10.1002/eap.2566)

Supporting Information. Patrick M. Garvey, Alistair S. Glen, Mick N. Clout, Margaret Nichols, and Roger P. Pech. Niche partitioning in a guild of invasive mammalian predators. Ecological Applications.

Appendix S1

Table S1. Native and introduced bird species recorded on camera traps

**New Zealand native species**	
<u>*Common name*</u>	<u>*Scientific name*</u>
Fantail	*Rhipidura fuliginosa*
Field pipit	*Anthus novaeseelandiae*
Grey warbler	*Gerygone igata*
Kingfisher	*Todiramphus sanctus vagans*
Morepork	*Ninox novaeseelandiae*
North Island robin	*Petroica longipes*
Paradise duck	*Tadorna variegata*
Rifleman	*Acanthisitta chloris*
Silvereye	*Zosterops lateralis*
Spur-wing plover	*Vanellus miles*
Swamp harrier	*Circus approximans*
Tom-tit	*Petroica macrocephala*
Tui	*Prosthemadera novaeseelandiae*
**Introduced species**	
Australian magpie	*Gymnorhina tibicen*
Blackbird	*Turdus merula*
California quail	*Callipepla californica*
Chaffinch	*Fringilla coelebs*
Common redpoll	*Carduelis flammea*
Goldfinch	*Carduelis carduelis*
Greenfinch	*Carduelis chloris*
Hedge sparrow	*Prunella modularis*
Skylark	*Alauda arvenis*
Song thrush	*Turdus hilomelos*
Sparrow	*Passer domesticus*
Starling	*Sturnus vulgaris*
Wild turkey	*Meleagris gallopavo*
Yellowhammer	*Emberiza citrinella*

Table S2. Naïve site use estimates (ψ(obs)) for predators (cat, ferret, stoat) over three monitoring periods (pre-control, post-control, 6 mth post-control). Site use estimates (ψ(.)) are included where predator detections were sufficient to estimate occupancy.

Predator	Recording period	Site	ψ(obs)	ψ (.)	SEψ(.)
Cat	Pre-control	Non-treatment	0.54	0.63	0.12
Cat	Post-control	Non-treatment	0.62	0.76	0.11
Cat	6 mth post-control	Non-treatment	0.49	0.86	0.22
Cat	Pre-control	Treatment	0.33	0.51	0.17
Cat	Post-control	Treatment	0.06	-	-
Cat	6 mth post-control	Treatment	0.08	-	-
Ferret	Pre-control	Non-treatment	0.05	-	-
Ferret	Post-control	Non-treatment	0.08	-	-
Ferret	6 mth post-control	Non-treatment	0.03	-	-
Ferret	Pre-control	Treatment	0.33	0.51	0.37
Ferret	Post-control	Treatment	0.09	-	-
Ferret	6 mth post-control	Treatment	0.06	-	-
Stoat	Pre-control	Non-treatment	0.08	-	-
Stoat	Post-control	Non-treatment	0.08	-	-
Stoat	6 mth post-control	Non-treatment	0.12	-	-
Stoat	Pre-control	Treatment	0.00	-	-
Stoat	Post-control	Treatment	0.03	-	-
Stoat	6 mth post-control	Treatment	0.14	-	-

Table S3. Parameter estimates of occupancy (ψ) and detection probability (*p*) for predators (cats, ferrets, stoats) and prey (rodents, lagomorphs, birds) during a three-weeks survey in Hawke's Bay, New Zealand. Trail cameras recorded target species prior to a pulse perturbation at two sites (treatment and control). The parameter ψ(obs) is the proportion of sites where a species was detected but is unadjusted for detection probability (i.e., naïve occupancy).

**Species**	**Study site**	**ψ(obs)**	**ψ(.)**	**SEψ(.)**	***p*(.)**	**SE*p*(.)**
Predator						
Cat	Control	0.54	0.63	0.12	0.32	0.07
Cat	Treatment	0.33	0.51	0.17	0.21	0.17
Ferret	Control	0.05	-	-	-	-
Ferret	Treatment	0.33	0.51	0.37	0.37	0.09
Stoat	Control	0.08	-	-	-	-
Stoat	Treatment	0.00	-	-	-	-
Prey						
Rodent	Control	0.44	0.51	0.09	0.46	0.07
Rodent	Treatment	0.11	-	-	-	-
Lagomorph	Control	0.15	0.17	0.07	0.4	0.12
Lagomorph	Treatment	0.49	0.53	0.06	0.45	0.09
Bird	Control	0.48	0.59	0.11	0.36	0.07
Bird	Treatment	0.62	0.72	0.12	0.41	0.07

Figure S1. Cat with rodent recorded using a camera trap at the treatment site in May 2014.

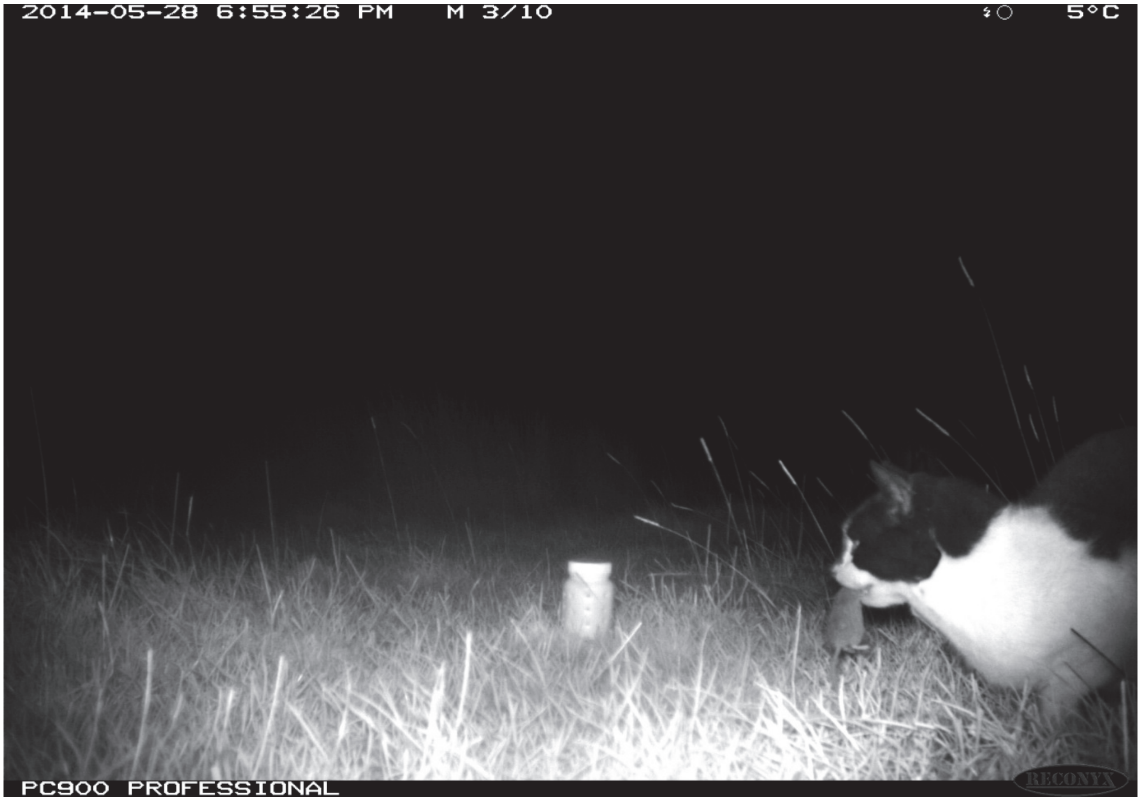

Figure S2. Stoat, with a dead rabbit, investigating the lure vial at the non-treatment site (recorded using a camera trap in November 2014)

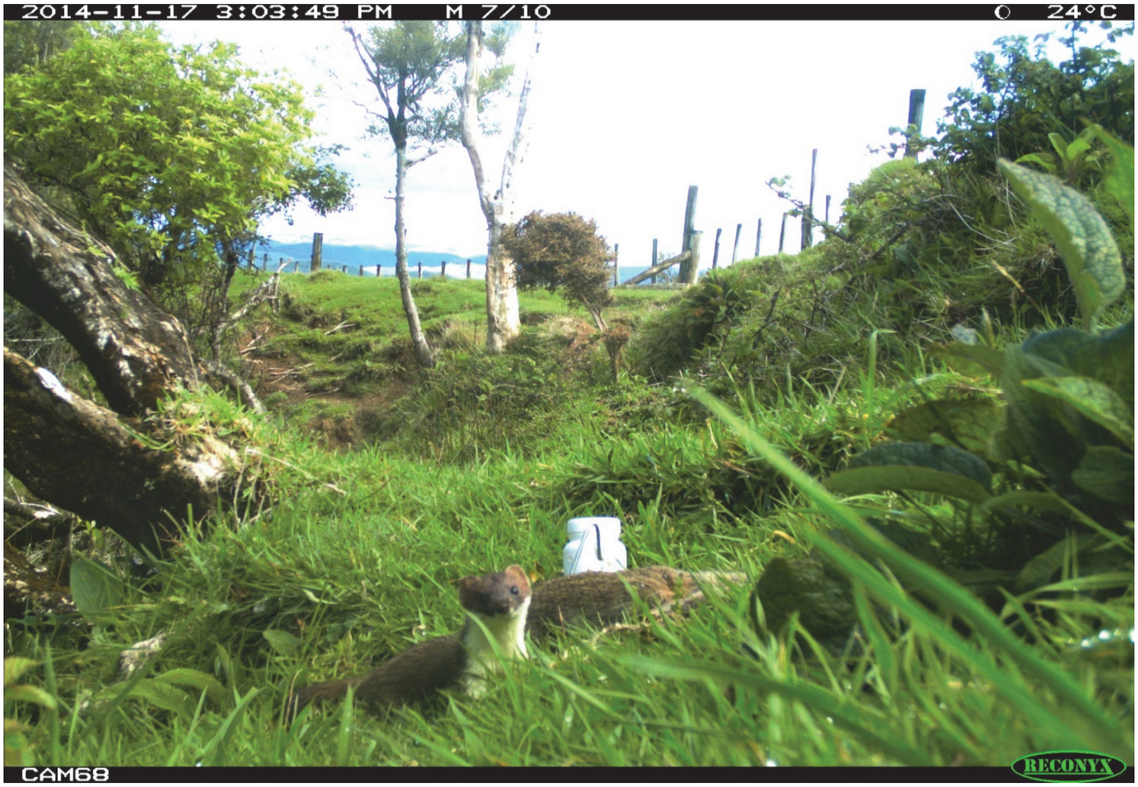

Supplement: Supplementary file 1 — Appendix S1 [file EAP-32-0-s001.pdf]
